# How Many Sexes, How Many Genders? And What Does This Imply for (Social) Scientists?

**DOI:** 10.1007/s10508-025-03191-6

**Published:** 2025-09-05

**Authors:** Amanda Klysing, Melanie C. Steffens

**Affiliations:** 1 Department of Psychology, Lund University, Box 213, 221 00 Lund, Sweden; 2 Department of Social, Environmental, and Economic Psychology, Faculty of Psychology, RPTU University Kaiserslautern-Landau, 76829 Landau, Germany

In some ways, it can feel like conducting empirical research has become more and more difficult. Some standards that were widely agreed upon in the past for conducting research on gender and/or sex appear to be contested today (see Fraser, 2018). Some questions that may feel uncontroversial to ask for some research participants are a source of minority stress, exclusion, and outrage for others. Can you, as a researcher, even ask for what you think is basic demographic information without revealing certain attitudes and risking to step on someone’s toes? The short response is: Probably not.

This short response requires some explanations about what makes gender, sex, and sexuality so intimately intwined, and why it is such a contested topic. And importantly: How can we as social scientists navigate this contested field in an informed and purposeful way?

## Biology: More Than Two Sexes

If a researcher asks their participants to indicate their sex, they are asking people about their bodies. That could be the intention (e.g., in a pharma test whether a new drug should be helpful for all sexes), but in many social science studies, it is not really what we are interested in (see, e.g., Lindqvist et al., 2020; Westbrook, 2015). Moreover, in children before puberty, because sexual characteristics are not yet fully developed, it is even possibly unknown what a participant’s sex is (i.e., whether their sex does completely fall into one of the two categories, male/female). Sex is often referred to as “female, male”—in the same way for many laboratory animals as for human participants.

The view that there are only two sexes, male and female, is outdated and too narrow as it ignores a bounty of evidence that sex is not a binary and dichotomous property (Hyde et al., 2019). People with variable sex characteristics (VSC)/intersex exist in bodies that are not exclusively male or female, and the large diversity of physical characteristics within the group further shows how binary sex does not accurately describe human heterogeneity (Hegarty, 2023). 

Scientists—along with politicians and activists—who claim that “there are only two sexes” have obviously forgotten this basic knowledge of biology. Thus, if one is interested in participants’ sex, it is important to include at least a third category for VSC/intersex people, making sure that all participants can correctly categorize themselves. Furthermore, there is great diversity of sexual characteristics within these sex categories, potentially making the very question too blunt to be truly informative.

This is because sex is not a simple dimorphic categorization: It is better conceived as a categorization based on family resemblance (Fox, 2011; Klysing, 2023b). “Sex” as a category is a conglomerate of different biological aspects where people in the same sex category share at least one biological element with each other, but not necessarily all of the elements (Hyde et al., 2019). Importantly, people can have elements from the “other” category; for example, a given woman can have a higher level of testosterone than a given man (Hyde et al., 2019). So rather than ask participants about their sex category as a shorthand for a biological aspect the research is interested in, it is more precise to ask about that specific aspect.

## Gender

But is even sex a simple biological, essential category devoid of social and cultural influences? How do we determine what are the crucial elements of a sex category and why is there so much policing around maintaining strict sex divisions if it is a natural category? As one striking example, medical interventions on infants to “normalize” their sex demonstrate that sex is not pure biology, but subject to cultural influences (Hegarty et al., 2021). The great majority of adults further engages in practices to change their bodies to better align with ideals for female or male bodies, such as dress, hair, use of cosmetics, plastic surgery, diet, and exercise regimes (e.g., Gruber et al., 2024). According to the performative theory of gender, this happens because there is no such thing as pre-cultural sex (Butler, 2006). Instead, the way that we create and make sense of sex is through appeal to gendered acts. When people act as women or men and use the concept of gender to understand the actions of others, it creates the very categories that these acts are supposedly based on (Butler, 2006). And because sex is a human-made categorization, our understanding of sexed categories is as cultural as gender is (Butler, 2011; Magnusson, 2011; Morgenroth & Ryan, 2018). A performative theory of gender implies that gender is not an internal thing that you have, but rather it is something that you do and something that emerges through interactions with other people (Butler, 2006; West & Zimmerman, 1987). However, most people still generally feel like they have a gender, which leads us to the topic of gender identity.

## Gender Identity: A Continuum

Gender identity is a complex concept that refers to the way an individual perceives and identifies themselves (see Fausto-Sterling, 2019). In a binary gender system, only two gender identities are possible: woman and man. However, when examining how people across different times and cultures perceive themselves, it becomes clear that gender identity can take many forms. Examples are identifying as being outside of the gender binary, as being in between woman and man, as shifting between gender identities, as not having a gender identity, or identifying as a different gender than woman or man (Hegarty et al., 2018). Individual gender identity is influenced by social and cultural factors, such as gender stereotypes, traditionally masculine and feminine social roles, and an individual’s sense of belonging to a gender group (Keener, 2015). Based on the interplay of the social and cultural factors and own identification, an individual actively forms a gender identity—as man, woman, as non-binary, as something else, or as none of the above.

All social categories with which people identify and to which people (want to) belong do strongly influence them in any case (see the social identity approach; e.g., Brown, 2020). But the gender binary that prevails in our culture is one of the strongest social categorizations (one of the Big Three, along with age and ethnicity/race). Moreover, the gender binary has been theorized to be the very basis of the Big Two in social perception: agency and communion (Martin & Slepian, 2021), thus making it the primary social categorization. It is also the one categorization that is, in many languages, hard to avoid (e.g., through specific pronoun use). Bem (1993) argued that gender is so pervasive in our culture because of three factors: biological essentialism, androcentrism, and gender polarization. We’ve already touched on how gender gets the status of a natural category through appeals to biological essentialism. Androcentrism is a pervasive social ideology that sees men as the norm for humanity, while non-men are seen as uniquely gendered beings (e.g., Hegarty et al., 2010). Gender polarization is both a consequence of and a contributor to the first two lenses: We divide the social world by gender throughout a majority of aspects of social life. Writing about “the opposite sex” is an instance of polarization (and should be avoided, according to current APA style). Blatantly put, to the best of our knowledge, all existing cultures are “Is it a boy or a girl?” cultures. Norms regarding gender have changed across time and differ between locations, but this differentiating between gender categories remains as the only stable meaning of gender (Hare-Mustin & Marecek, 1988; Magnusson, 2011).

In short, gender is ubiquitous, visible, and enacted permanently. From infancy on, gender influences our perception of ourselves, our interests, our abilities, and our behavior (Leaper & Friedman, 2007). Gender is also hard to avoid linguistically, especially in languages such as German where every noun has a gender (for a review, see Stahlberg et al., 2007). The language we use can contribute to the invisibilization and invalidation of women and non-binary people. Psychological experiments have convincingly demonstrated that the use of so-called generic masculine noun forms, such as “Lehrer” (teacher in German), is not internally represented as generic, but introduce a male bias, making women and non-binary people invisible. Many social scientists and feminists have started using adapted language (such as “Lehrer*innen” for teachers who could be male, non-binary, or female). This has recently been prohibited in official documents in the German federal state of Bavaria—demonstrating political influence on the use of gender-related language in Europe (https://www.br.de/nachrichten/bayern/bayern-beschliesst-verbot-von-gendersprache,U7T9VzC).

Taken together: Because there is no such thing as pure biological sex in our culture, it has been recommended to refer to “gender/sex” (Unger & Crawford, 1993), which could work as an imprecise but rather inclusive choice in a questionnaire. This combination of options is, however, likely to read for a participant as an expectation that their gender/sex is aligned in a normative way. Again, the question asked should be related to what you want to know. For a research question about gender as a social identity, it may be most straightforward to ask what gender group someone belongs to. If needed, a second question about sex assigned at birth can be added, as can a question about current legal gender.

## Sexual Orientation and Gender Identity

Sexual orientation is another complex concept that refers to the way an individual is attracted to other people. Sexual orientation cannot be separated from gender identity, as they are closely linked: If we lived in a culture in which gender was not important, but sex was only one means of categorizing people next to many others, the construct of sexual orientation would be meaningless. If we lived in a culture devoid of gender polarization, how could a person’s sexual orientation even be defined?

Binary gender is justified by the existence of a heterosexual matrix of cultural intelligibility (Butler, 2006). Heterosexuality is seen as the natural state of human existence with reference to the fact that humans are sexually reproducing creatures. However, sexual reproduction does not require the social institution of heterosexuality that is socially enacted with strict gender roles and a power imbalance between men and women (Connell, 1987). To make the social structure of heterosexuality seem natural, humanity is divided into two differing, dichotomous, and complementary groups that can only be complete in relationship with each other (Butler, 2006; Morgenroth & Ryan, 2021). People who live outside of heterosexuality therefore do not fit into this binary gender system. This can be seen in the way that stereotypes about sexual minorities are gender inverted (Klysing et al., 2021; Mize & Manago, 2018), that only heterosexual women and men are seen as prototypical women and men (Klysing, 2023a), or that leading theories around the “cause” of homosexuality (not heterosexuality!) stipulate that it is due to abnormal levels of fetal androgens making female fetuses male-typed and male fetuses female-typed (Rosario & Schrimshaw, 2013). In a sense, rejecting the hegemony of heterosexuality removes people from the binary gender system as well (Wittig, 1981), making all sexual minorities (and those enacting a non-normative heterosexuality) non-binary in a type of way.

Due to the intricate nature of gender and sexual orientation, we need to be careful and intentional in our research. We need to be mindful that when we speak about, for instance, women or men in general, we are likely not speaking to the experiences of sexual minorities. For example, the widely used Ambivalent Sexism Inventory contains items such as “Every man ought to have a woman whom he adores” (Glick & Fiske, 1997), which neglects non-heterosexual experiences. As one consequence questionnaire responses may not make sense (what should I tick if I think every man ought to have a partner whom he adores?). Another consequence is that to participants who find their identities and experiences invalidated such research could feel like a microaggression (see Fattoracci et al., 2021). And a third consequence is that a researcher cannot simply use such a validated questionnaire without invalidating non-heterosexual experiences. The general point is that researchers should always try to be inclusive and clear (e.g., an advance instruction could help: “The following statements refer to heterosexual relationships”). 

On the flip side, when we conduct research on sexual minorities, it is important to keep gender in mind, as minority groups will have experiences in common due to their sexuality but also different experiences due to their genders (Klysing et al., 2024). Furthermore, gender identity and gender expression within the LGBTQI+ community are infinitely diverse and multifaceted (Levitt, 2019), so using conceptual frameworks from outside of the community is likely to lead to poor understanding of the research—and often to anger and outrage, too. It may help to involve community members in the conceptualization and pretesting of a study.

Exactly how to involve members of a community will depend on the features of the research project itself (Vaughn & Jacquez, 2020). There are several existing guidelines on performing participatory research that can be helpful in adapting community involvement to best inform the research (Duea et al., 2022). One example is to establish a community advisory board consisting of community members with different relationships to the community that is involved in the research design, analysis, and dissemination (Duea et al., 2022). These practices are an important part of conducting ethical, inclusive research that can inform policy and practice (Henrickson et al., 2020).

## Why Is There a Clash of Cultures and How Do We Navigate It?

What makes gender/sex-related categories so contested? How come the way we talk about gender/sex provokes fierce debate in the political arena? In short, our ideas are that there are different views; that it is complicated and subject to change (nothing we write here might stand the test of time); and that it is a discussion involving many aspects of power.

We first address the points that this topic is complicated and that there are several different views to account for. As one instance, different expressions stress different things—for example, using the question “Do you identify as…”: This implies subjectivity, maybe even choice. Regarding sexual orientation, this phrase is acceptable for many. After all, researchers’ choices are to either categorize people based on behavior (“men who have sex with men”), completely disregarding whether they identify as straight, bi, gay, or none of these terms. Or we ask for subjective sexual orientation because not all sexual orientations correspond to a specific behavior; for instance, a woman who has chosen only to have relationships with women could still have a bisexual identity. When making this choice to ask for behavior or identity, it is important to stick to it when you describe your participants. If the data that have been collected are on sexual behavior, one needs to avoid ascribing sexual identities.

Regarding gender, non-binary individuals may on the one hand object to the expression “identify as,” stressing rather “is” because they feel the validity of their being non-binary is otherwise questioned. On the other hand, many genderqueer people object to the term “is” because it implies a fixed and internal identity rather than a rejection of being defined (Roselló Peñaloza & Cabruja Ubach, 2015; Worthen, 2022). Explaining to participants why a certain term is chosen can potentially help. A complementary way is using participatory methods to ground one’s research within the community of interest (Klysing et al., 2024)

As a second example of “complicated,” the term “gay” is frequently used as an umbrella term but is imprecise; it is unclear whether one refers to men or also women. (As has become clear, it is difficult to conceptualize at all if there is no gender binary.) Therefore, the expressions “gay men” and “lesbian women” are recommended by the APA. However, some lesbians object against being called “wo-man,” reminiscent of a derivative of “man” or because they do not feel connected to the heterosexualized concept of woman. In several languages, the term homosexual is used as a relatively neutral umbrella term for lesbians and gay men (e.g., in Swedish), while current APA guidelines ask researchers to avoid this term due to its pathologizing history. Sexual identity terms like gay or lesbian will also not be suitable for all cultural contexts, where people may conceptualize their own sexuality and gender in different ways (Klysing et al., 2024).

Another important point to consider is that gender is an arena of power. Gender structures are a way to create and control reproductive subjects, to build society in line with dominant ideologies (Connell, 1987; Pratto & Walker, 2004). In current days, gender has also become a focus point for several different concerns about the state of the world, with fears caused by civil unrest and changing social dynamics attributed to “gender ideology” (Butler, 2024). Within anti-gender ideology, gender has become a phantasm that makes subjective reality into actual reality. These fears are performative, in the sense that feelings of threat to the social order become felt as actual threats to the stability of the world (Butler, 2024). More generally, social identity threat results if social categories are contested that are important to individuals (Branscombe et al., 1999; Ehrke & Steffens, 2025; Steffens et al., 2017). Most people are highly identified with gender categories, so it is not surprising that discussing these categories creates threat (Morgenroth & Ryan, 2021).

For researchers within social sciences, it is important to remember that the supposed social order of the past with unthreatened patriarchal power and global stability and prosperity is merely a dream: There has never been a world without human strife and gender trouble. The social sciences are intrinsically linked to the politics of the day, with our epistemologies, theories, methodologies, and research findings influenced by our existence as societal subjects (Cherry, 1995; Gergen, 1973). So how can we be aware of the ideological positions that our choices as researchers are based on? And how can we work toward a social science that creates truthful knowledge and is a force that promotes the equal value of all people?

Maybe one thing has changed across time. Some decades ago, researchers thought it was okay to exclude/invalidate participants if only a small percentage was excluded (e.g., if 98% of research participants identified as man or woman). But today, there is higher awareness of the microaggressions this implies for those being miscategorized or invalidated—especially as their vulnerability is often high because of painful past experiences.

To avoid excluding marginalized groups from our research, we can expand our methodological toolbox. The quantitative methods that require a certain number of participants in each group have many benefits, but when they are used to justify the exclusion of minorities from entire research fields, they cannot stand alone. Quantitative methods can be particularly poor at informing important questions about how different social categories influence each other, with intersectional experiences often requiring a deeper look at specific positions than afforded by group level analyses (Bowleg, 2008; Cole, 2009; Else-Quest & Hyde, 2015; McCall, 2005; Settles et al., 2020; for a recent experiment, see Ball et al., 2025). A social science that is informed about different epistemologies and the methodological toolboxes they come with will be better served to make informed decisions and foresee the consequences of different ways of doing research.

So, what to do in your study? Decide what type of knowledge you want to contribute. Get informed about the types of questions that different methodologies can answer. Decide what methodological tool is most adequate for your purpose, what knowledge it can and cannot create. Decide whom to address. And decide whom you are willing to lose as a participant because they are outraged about your using a certain term (e.g., sex, gender, or gender/sex) and a certain number of categories (at one extreme, only three gender/sex categories; at the other, a long list of categories).

## Conclusion: Toward Solving the Gender Dilemma

Ideally, we could leave the “Is it a boy or a girl?” culture behind. A person’s sex would play a role in, for instance, breeding, breast-feeding, and drug test trials where biological differences between bodies are important. But not in kindergarten, not when deciding whether to study engineering or psychology, not on the job. This expansion of the reproductive arena to areas of life where it is not relevant only paves the way for continued social inequality (Connell, 1987). Gender blindness is often better for women (e.g., Martin & Phillips, 2017); women may choose not to identify with gender (see Munzer et al., 2025); and as we have discussed, the prototypes of women and men are heterosexual (and White), excluding many people with intersecting identities. In that way, US president Donald Trump’s political agenda and Swedish kindergartens that avoid gender pronouns ironically align quite well in several respects. But while an informed gender blindness can serve the interests of marginalized groups, the current anti-gender ideologies of conservative movements are at their core fundamentally about preserving a deeply gendered world.

In the long term, it would be great if we could do without the term “gender.” However, the “Is it a boy or a girl?” culture is currently alive and kicking. As social scientists, the terms we choose to use will inevitably signal how we conceptualize gender as a phenomenon in the world: as gender, as sex, as gender/sex. While this is the case, it is important to signal that we are welcoming, and to stop invalidating non-binary people (by stating more pronouns than ever before); to use gender quota to mitigate hurdles on women’s way to the top; to provide shelters and support structures specifically for women; and to signal openness to different sexual orientations. At the same time, we are hoping that all this is temporary, while moving toward cultures that overcome the gender/sex binary (rendering the concept of sexual orientation meaningless), where each individual is free to develop their talents and interests without being held back by gender stereotypes.
